# Ipsilateral concomitant fractures of the clavicle and coracoid process of the scapula: incidence, characteristics, and outcomes

**DOI:** 10.1186/s10195-025-00817-2

**Published:** 2025-01-18

**Authors:** Nan Zhang, Guoyang Bai, Xiaomin Kang, Yangjun Zhu, Dongxu Feng

**Affiliations:** 1 Department of Pathology, Hong Hui Hospital, Xi’an Jiaotong University School of Medicine, Xi’an, 710054 Shaanxi China; 2 Shaanxi University of Chinese Medicine, Xi’an, 712046 Shaanxi China; 3 Center for Translational Medicine, the First Affiliated Hospital of Xi’an Jiaotong University, Xi’an, 710061 Shaanxi China; 4 Department of Orthopaedic Trauma, Hong Hui Hospital, Xi’an Jiaotong University School of Medicine, Xi’an, 710054 Shaanxi China

**Keywords:** Clavicle, Coracoid process, Fractures, Open reduction and internal fixation, Superior shoulder suspensory complex

## Abstract

**Background:**

Clavicle fractures associated with ipsilateral coracoid process fractures are very rare, with limited literature reporting only a few cases. This study reports on 27 patients with ipsilateral concomitant fractures of the clavicle and coracoid process who were followed for more than 12 months.

**Material and methods:**

This retrospective study reviewed the charts of skeletally mature patients with traumatic ipsilateral clavicle and coracoid process fractures treated at the authors’ institution. Each patient was regularly followed post-treatment. Radiographs assessed bone union and implant integrity, while clinical evaluations included the Constant–Murley score for shoulder function; disability of the arm, shoulder, and hand (DASH) questionnaire for upper limb function; and visual analog scale score for pain. Complications were also recorded.

**Results:**

From October 2012 to February 2023, 40 patients were diagnosed with ipsilateral fractures of the clavicle and coracoid process of the scapula, accounting for 1.4% (40/2877) of all clavicle fractures and 5.2% (40/786) of all scapular fractures. This study included 27 patients with follow-up exceeding 12 months: 6 had medial-third clavicle fractures, 12 had middle-third fractures, and 9 had distal-third fractures. According to Eyres’ classification, the coracoid fractures included two type I, five type II, eight type III, seven type IV, and five type V fractures. Twenty-two patients received operative treatment, with clavicle fractures fixed with internal plating and 11 coracoid fractures with internal fixation. Bone union was achieved in all patients. The mean Constant–Murley score was 91.2 ± 9.4 and the mean DASH score was 6.4 ± 7.6. Five patients reported mild shoulder pain and five patients developed complications.

**Conclusions:**

Ipsilateral concomitant fractures of the clavicle and coracoid process can occur at various clavicle locations, with shaft and medial fractures more common than previously thought. Displaced fractures can be effectively managed with operative treatment, and coracoid process fixation may not be necessary if satisfactory indirect reduction is achieved after clavicle fixation.

*Level of evidence*: Level III, retrospective cohort study.

## Introduction

Clavicle fractures are among the most common fractures of the shoulder girdle, comprising 2.6–10.0% of all fractures [1], with many cases occurring in young adults. Fractures of the middle-third of the clavicle are most frequent, accounting for 80% of clavicle fractures, while lateral and medial clavicle fractures are less common, representing 17–20% and 3% of cases, respectively [2, 3]. The primary mechanisms of clavicle fractures include simple falls on the shoulder (31%), traffic accidents (27%), and sports injuries (23%) [3]. Additionally, indirect trauma, such as falling on an outstretched arm, is more common than direct impact trauma. Middle clavicle fractures often occur in young patients with high-energy injuries, while distal clavicle fractures are more common in elderly patients with osteoporosis. Medial clavicle fractures are frequently associated with high-energy trauma or multiple injuries [4].

A fracture of the coracoid process of the scapula is uncommon, accounting for approximately 3–13% of all scapular fractures [5]. Scapular fractures themselves are rare, making up only 1% of all fractures and 5% of shoulder fractures [6]. Coracoid process fractures are typically caused by high-energy trauma and are often part of complex shoulder girdle injuries [7, 8].

In some cases, injury may involve both clavicle fractures and ipsilateral coracoid process fractures; however, literature on such injuries is limited, with only a few cases reported [6, 9–12]. This study aimed to report the incidence, characteristics, and clinical treatment outcomes of concomitant clavicle fractures and ipsilateral coracoid process fractures.

## Materials and methods

### Ethics statement

This study was approved by the Ethical Committee of our hospital (no. 202208002), and performed in accordance with the 1964 Declaration of Helsinki and its later amendments or comparable ethical standards. All patients signed written informed consent forms and agreed to publish their images for medical research.

### Study design and participants

The clinical data of all skeletally mature patients with traumatic clavicle or scapular fractures treated at our institute from October 2012 to February 2023 were retrospectively reviewed. Inclusion criteria were skeletally mature patients with both clavicle and ipsilateral coracoid process fractures and a follow-up period exceeding 12 months. The exclusion criteria were pathological fractures, a simple isolated clavicle or coracoid process fracture, coracoid process fractures with acromioclavicular or sternoclavicular joint dislocation, or fractures of the clavicle and coracoid located on different sides.

Patients’ demographic data, injury mechanisms, fracture patterns, comorbidities, and concomitant injuries were collected from the medical record system. Clavicle fractures were divided into medial-third, middle-third (shaft), and distal-third fractures according to the Allman classification [13]. The Edinburgh classification [14] and Neer’s classification [15] were subsequently used to categorize medial and distal clavicle fractures, respectively. Coracoid process fractures were categorized as type I (tip or epiphyseal fracture), type II (mid-process fracture), type III (basal fracture), type IV (superior body of scapula involved), or type V (extension into the glenoid fossa) on the basis of Eyres’ classification [16]. Concomitant sternoclavicular joint dislocations were classified as anterior dislocation or posterior dislocation [17], while acromioclavicular joint dislocations were categorized into six types according to the Rockwood classification [18].

### Surgical technique

**Fig. 1:**
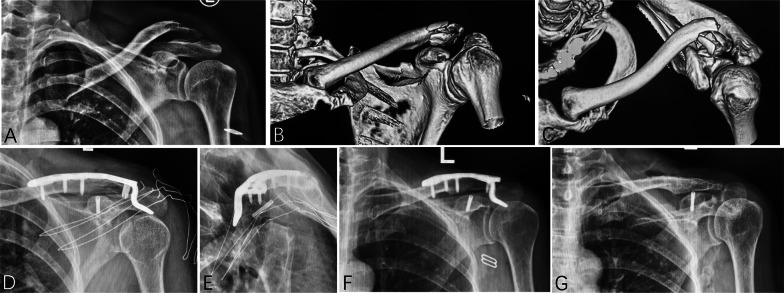
A 56-year-old woman (patient 3) was injured by a car while crossing the road. Preoperative **A** X-ray and **B**, **C** computed tomography (CT) scan showed a displaced comminuted distal clavicle fracture combined with ipsilateral Eyres type IV coracoid process fracture. **D**, **E** The distal clavicle fracture was fixed with a clavicle hook plate, and the coracoid process was fixed with a headless screw. **F** X-ray taken 20 months postoperatively showed solid bone union with no implant failure, and the patient was very satisfied with the treatment and returned to her pre-injury work. **G** She later underwent implant removal

**Fig. 2:**
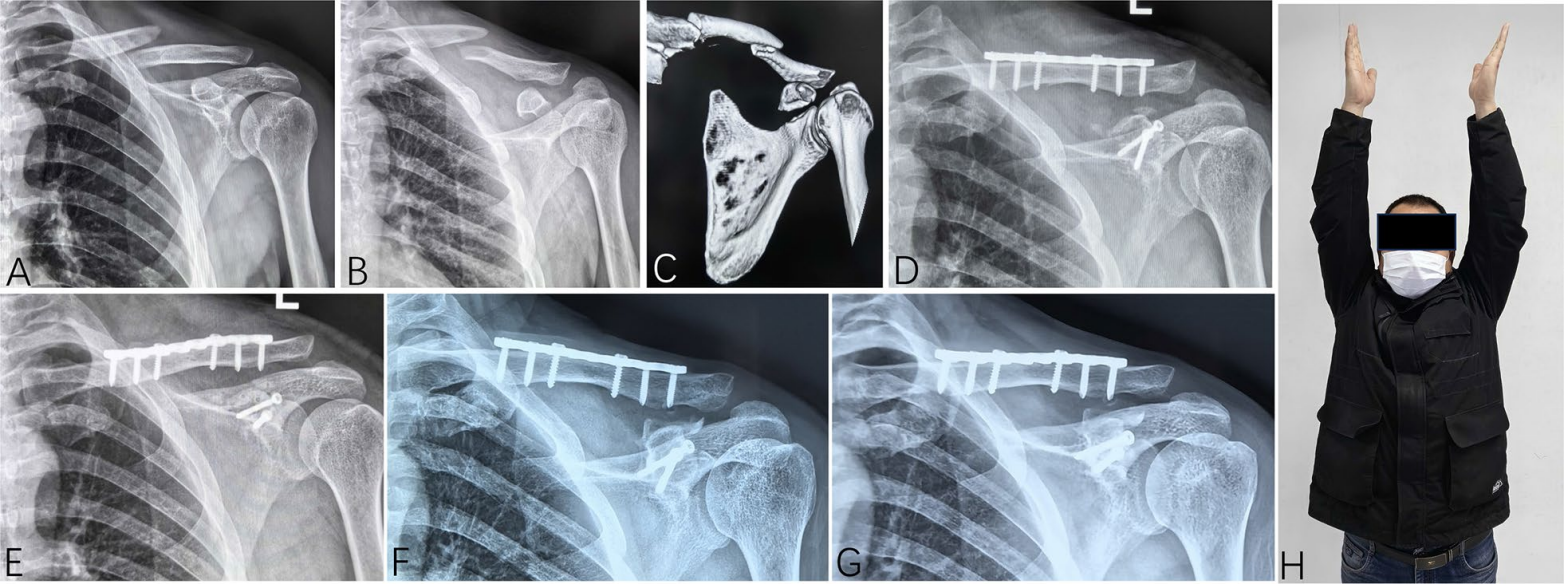
A 43-year-old man (patient 23) sustained a displaced clavicle shaft fracture and a displaced ipsilateral Eyres type III coracoid process fracture due to a fall from height. **A**–**C** Preoperative imaging showed the fracture. **D**, **E** He was treated with open reduction and internal fixation. **F**, **G** X-rays taken 12 months postoperatively showed solid bone union with no implant failure. **H** He achieved a full range of shoulder motion and returned to his pre-injury work

After administration of a regional interscalene block and general anesthesia, the patient was placed in a semi-sitting position on a radiolucent table with a large “bump” between the scapulae to allow passive mobilization of the injured shoulder intraoperatively. An incision was made along the superior aspect of the clavicle, centered over the fracture, with careful identification and preservation of the supraclavicular nerve where possible. After debridement, the fracture was reduced and temporarily fixed with Kirschner wires, followed by fixation of the fracture fragments with an appropriate plate (either a superior locking plate or a clavicle hook plate). The position of the plate and length of the screws were checked by intraoperative fluoroscopy. If satisfactory indirect reduction of the coracoid process was achieved after clavicle fixation, further fixation of the coracoid was unnecessary. If not, the anterior portion of the deltoid was released from the clavicle to expose the coracoid, and under direct visualization, the coracoid fracture was reduced and fixed with suitable implants (Figs. 1 and 2). In some cases, a robot-assisted technique was used to guide screw direction and length. The wound was closed in layers.

### Postoperative management and evaluation

Patients treated operatively were immobilized with a sling for 3 weeks postoperatively and were encouraged to begin gentle passive shoulder abduction, forward flexion, and horizontal rotation exercises immediately after surgery under the supervision of a physiotherapist, as well as active elbow flexion and extension exercises. Codman’s pendulum exercises and active-assisted motions were initiated at 3 weeks postoperatively. Active strengthening exercises were initiated at 3 months postoperatively. Normal activity and sports exercises were allowed at 6 months postoperatively following bone healing.

A sling was applied for 4 weeks to each patient who received conservative treatment. Codman’s pendulum exercises were then gently initiated, and patients gradually began passive shoulder forward flexion, abduction, and horizontal rotation exercises. At 6 weeks postoperatively, active range of motion exercises were started. Similar to the above, active strengthening exercises began at 3 months, and normal activity and sports exercises were allowed after bone healing.

Each patient was followed up at 1, 2, 3, and 6 months postoperatively, and every 6 months thereafter or until bone healing. Radiographs and clinical evaluations were performed at each follow-up. Fracture union was defined as evidence of at least three of four healed cortices across the fracture site. The Constant–Murley score was used to evaluate shoulder function, with scores between 86 and 100 points considered excellent, 71 to 85 points as good, 56 to 70 points as fair, and less than 56 points as poor [19]. The disability of the arm, shoulder, and hand (DASH) questionnaire was used to assess upper limb function, with lower scores indicating higher limb function [20]. A visual analog scale (VAS) ranging from 0 to 10 was used to assess pain, with higher scores representing greater pain levels [21]. Any complications were also recorded.

### Statistical analysis

Results are presented as mean ± standard deviation (SD). Statistical evaluation of the data was carried out using SPSS Statistics for Windows, version 21.0 (IBM Corp., Armonk, NY, USA).

## Results

From October 2012 to February 2023, 2877 patients with clavicle fractures and 786 patients with scapular fractures were treated at the authors’ institute. Among these, 40 patients were diagnosed with concomitant ipsilateral fractures of the clavicle and coracoid process of the scapula, accounting for 1.4% (40/2877) of all clavicle fractures and 5.2% (40/786) of all scapular fractures.

A total of 27 patients had a follow-up period of longer than 12 months (Table 1), including 21 men and 6 women, with a mean age of 35.9 ± 13.6 years (range, 25–77 years). In total, 14 patients were injured on the left side, and 13 were injured on the right side. The injury mechanisms included car accidents (*n* = 10), falls from height (*n* = 10), crashes (*n* = 2), slips (*n* = 3), bicycle accidents (*n* = 1), and motorcycle accidents (*n* = 1). Nineteen patients (70.4%) had associated concomitant injuries, with chest injury being the most frequent (13/27). Six patients had medial-third clavicle fractures (one type 1A1, two type 1B1, and three type 1B2 according to the Edinburgh classification), 12 had middle-third (shaft) fractures, and 9 had distal-third fractures (four type I, two type II, and three type III on the basis of the Neer classification). According to Eyres’ classification, there were two type I, five type II, eight type III, seven type IV, and five type V coracoid process fractures. One patient had a concomitant anterior sternoclavicular joint dislocation, and four patients had acromioclavicular joint dislocations.

**Table 1:** Patients’ data

No.	Gender	Age	Side	Injury mechanism	Clavicle fracture classification	Coracoid fracture classification	Acromioclavicular joint dislocation	Associated injury	Treatment of clavicle	Treatment of coracoid	Follow up (months)	Shoulder flexion (°)	Constant–Murley	DASH	VSA	Complication
1	M	25	R	Car accident	Distal-third, type III	Type IV	Type III	None	Hook plating	One screw	21	170	95	7.5	0	None
2	M	43	L	Car accident	Distal-third, type III	Type III	–	Chest injury	Hook plating	One screw	18	165	96	3.3	0	None
3	F	56	L	Car accident	Distal-third, type III	Type IV	–	None	Hook plating	One screw	27	160	90	7.5	0	None
4	M	50	R	Fall from height	Medial-third, type 1B1	Type IV	–	Ipsilateral scapular fracture	Reversed distal anatomic locking plating	Two screws	25	165	92	5	0	Protrusion at medial clavicle
5	F	54	R	Fall from height	Middle-third	Type V	–	Contralateral femoral neck fracture	Superior plating	–	58	170	94	5	0	None
6	M	29	R	Fall from height	Medial-third, type 1B2 fracture – anterior dislocation	Type V	Type III	Chest injury, ipsilateral scapular fracture	Hook plating	One screw and one T-plate	31	155	90	6.7	0	None
7	M	47	L	Crashing	Middle-third	Type II	Type III	Contralateral clavicle fracture, chest injury	Primary surgeries in local hospital: plate for middle-third fracture and EndoButton for acromioclavicular joint dislocation Revision in our hospital: plate for middle-third fracture and hook plate acromioclavicular joint dislocation	–	17	60	54	35	2	Residual type II AC redislocation after implant removal 1 year postoperatively
8	M	30	L	Slipping	Distal-third, type II	Type II	–	None	Hook plating	–	43	170	94	3.3	0	None
9	F	36	L	Car accident	Middle-third	Type V	–	Chest injury, concussion	Superior plating	One screw	25	175	100	2.5	0	None
10	M	44	R	Car accident	Medial-third, type 1B2	Type II	–	Contralateral tibio-fibular fracture, pelvic fracture, lumbar fracture, ipsilateral shoulder dislocation	Nonoperative treatment	–	28	165	96	7.5	2	Protrusion at medial clavicle
11	M	41	R	Crashing	Medial-third, type 1B1	Type V	–	Brachial plexus injury, chest injury, acromion fracture	Locking plating	–	24	160	96	5	0	None
12	M	38	L	Fall from height	Distal-third, type II	Type IV	–	Chest injury	Hook plating	–	41	175	100	0	0	None
13	M	39	R	Car accident	Distal-third, type I	Type III	–	None	Hook plating	Two screws	62	160	92	2.5	0	None
14	F	35	R	Car accident	Middle-third	Type IV	–	None	Superior plating	Two Kirschner wires	37	170	92	2.5	0	None
15	M	67	L	Fall from height	Middle-third	Type III	–	Chest injury	Superior plating	Two screws	61	170	86	7.5	0	None
16	M	60	L	Bicycle accident	Middle-third	Type IV	–	Ipsilateral scapular fracture	Superior plating	-	53	150	86	5	0	None
17	F	25	R	Slipping	Distal-third, type I	Type IV	–	None	Hook plating	–	18	170	98	1.67	0	None
18	M	53	L	Motorcycle accident	Middle-third	Type III	–	Chest injury	Superior plating	–	16	150	90	5	1	None
19	M	64	R	Fall from height	Medial-third, type 1A1	Type II	–	Ipsilateral proximal humerus fracture	Nonoperative treatment	–	41	150	86	7.5	1	None
20	M	36	R	Fall from height	Medial-third, type 1B2	Type I	–	Ipsilateral open humeral intercondylar fracture, acetabular fracture, lumbar transverse process fracture, chest injury	Nonoperative treatment	–	20	170	94	3.3	0	None
21	M	61	L	Fall from height	Distal-third, type I	Type III	–	Ipsilateral proximal humeral fracture, chest injury, thoracic vertebral fractures, contralateral femoral neck fractures	Nonoperative treatment	–	93	150	88	5	0	None
22	M	60	R	Slipping	Middle-third	Type III	–	None	Superior plating	–	55	170	96	3.3	0	Residual type II AC dislocation, coracoid fracture missed diagnosed on preoperative images
23	M	43	L	Fall from height	Middle-third	Type III	–		Superior plating	Two screws	15	170	100	1.7	0	None
24	M	41	L	Car accident	Distal-third, type I	Type III	–	Ipsilateral humeral shaft fracture, contralateral clavicle fracture, chest injury, brain injury	Nonoperative treatment	–	109	165	98	1.7	0	None
25	F	77	L	Car accident	Middle-third	Type II	–	Ipsilateral toe bone fracture	Superior plating	–	28	145	73	27.5	2	None
26	M	31	L	Fall from height	Middle-third	Type V	Type II	Pelvic fracture, contralateral intertrochanteric fracture, chest injury	Superior plating	One screw	13	170	96	3.3	0	Residual type II AC dislocation
27	M	53	R	Car accident	Middle-third	Type I	–	Chest injury	Superior plating	–	24	160	90	5.8	1	None

Medial clavicle fractures were categorized according to the Edinburgh classification. Distal clavicle fractures were categorized according to the Neer’s classification. Acromioclavicular joint dislocations were categorized according to the Rockwood classification. *VAS* visual analog scale, *DASH score* disability of the arm, shoulder, and hand questionnaire score, *AC* acromioclavicular joint, *F* female, *M* male, *L* left, *R* right

Twenty-two patients were managed with operative treatment, while five patients were treated nonoperatively. Among the patients who underwent operative treatment, each clavicle fracture was fixed with an internal plate. Eleven coracoid process fractures were fixed with internal fixation (nine cases with screws, one case with two Kirschner wires, and one case with one screw and a T-shaped plate), and three coracoid process fractures were fixed using robot-assisted technology. Each patient achieved bone union at both the clavicle and coracoid process fracture sites during follow-up, and six patients underwent implant removal after bone healing. With a mean final follow-up of 31.1 ± 24.1 months (range, 13–109 months), the mean Constant–Murley score was 91.2 ± 9.4 points (range, 54–100 points, with 25 excellent cases, 1 good case, and 1 poor case) and the mean DASH score was 6.4 ± 7.6 points (range, 0–35 points). Five patients reported mild shoulder pain, and five patients developed complications (two cases of skin protrusion, three cases of residual acromioclavicular joint dislocation).

## Discussion

The coracoid process is a small, hook-shaped part of the scapula that projects anterolaterally from the superior aspect of the scapular neck. It serves as a critical anchor for various upper limb flexion muscles and ligament attachments. The coracoclavicular ligaments, which attach to the coracoid process, play a stabilizing role for the clavicle [5, 22]. Solitary fractures of the coracoid process are rare, and a displaced coracoid process fracture is often part of multiple shoulder injuries. In this study, one coracoid fracture (patient 22) was misdiagnosed on preoperative plain radiographs, possibly because of the overlap of bone structures at the coracoid process [23]. Thus, a computed tomography scan is necessary when there is high clinical suspicion of a coracoid process fracture. In 1996, Goss [24] noted that coracoid base fractures were the most common type, which is consistent with our findings, as a total of eight patients in this study sustained an Eyres type III (coracoid base) fracture.

As stated above, 40 patients in this study were diagnosed with concomitant ipsilateral fractures of the clavicle and coracoid process, accounting for 1.4% (40/2877) of all clavicle fractures and 5.2% (40/786) of all scapular fractures. Although clavicle fractures are common, fractures of the clavicle and coracoid process occurring simultaneously are rare, with limited literature reporting only a few cases. Doğar et al. [9] reported a 20-year-old man with concomitant fractures of the ipsilateral distal clavicle, clavicle shaft, Eyres type IV coracoid, and acromion. Wu et al. [6] managed a 21-year-old woman operatively with concomitant ipsilateral fractures of the coracoid, acromion, and distal clavicle. Nanno et al. [10] described a 38-year-old man with double clavicular fractures associated with fractures of the scapular neck and coracoid process. Sharma et al. [25] reported a 65-year-old man with a clavicle shaft fracture associated with ipsilateral acromioclavicular dislocation, coracoid fracture, and humeral shaft fracture.

By contrast, in a systematic review of coracoid process fractures, Ogawa et al. [26] found that 33 of 197 patients with coracoid process fractures also had clavicle fractures. In those cases, the clavicle fracture location was distal in 13 patients, midshaft in 7, unspecified in 11, both midshaft and distal in 1, and an avulsion of the inferior cortex in 1. For patients with both clavicle and coracoid process fractures, previous studies indicated that most clavicle fractures were located distally [8, 11, 16, 27]. Sun et al. [12] reported three cases of clavicle shaft fractures associated with ipsilateral Eyres type III coracoid fractures.

**Fig. 3:**
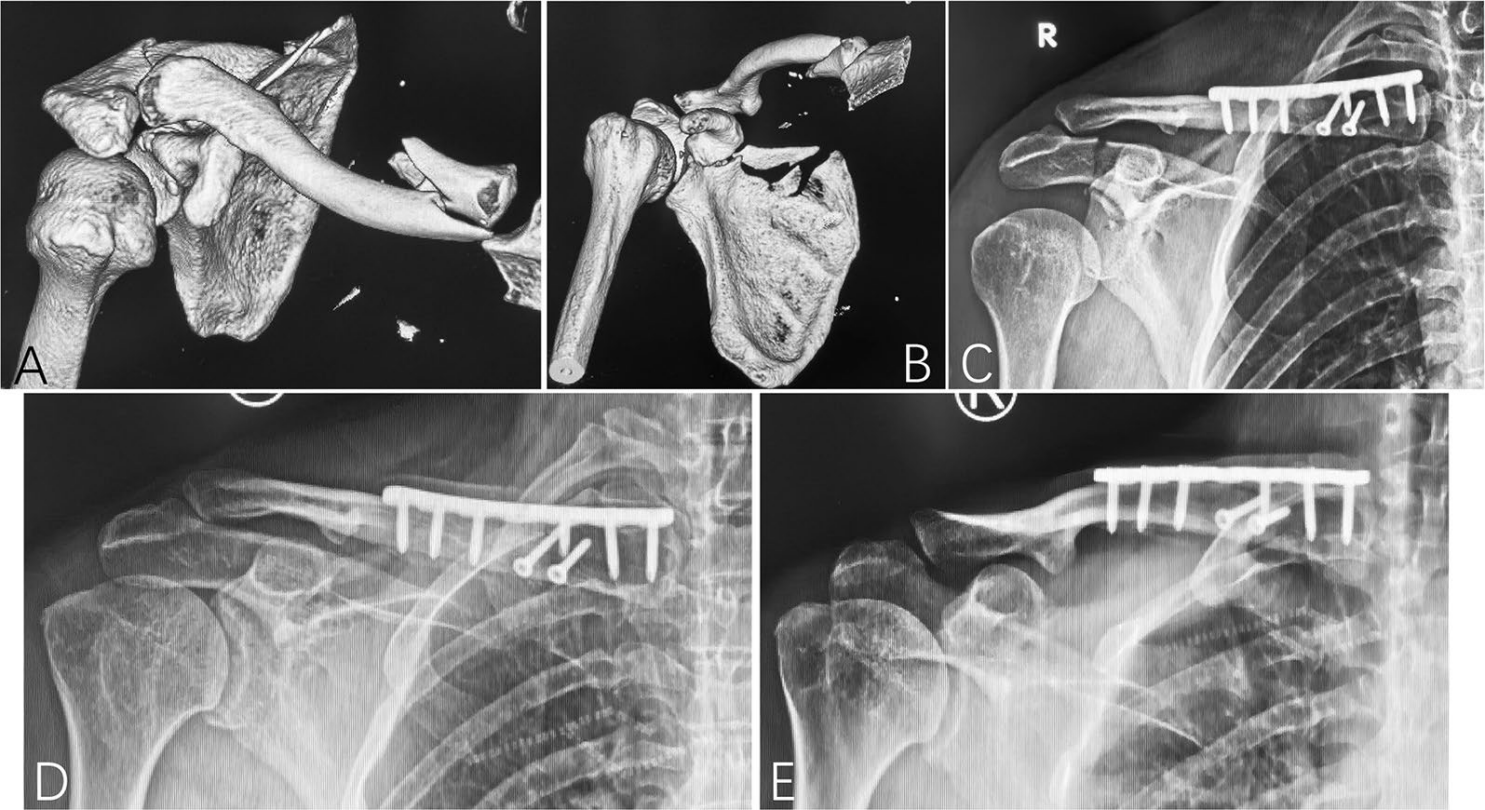
A 41-year-old male worker (patient 11) was injured by falling glass while at work. **A**, **B** He sustained a displaced medial clavicle fracture, an ipsilateral type V coracoid process fracture, an ipsilateral acromion fracture, transient brachial plexus injury, and chest injury. **C** The medial clavicle fracture was fixed with plating. **D, E** X-rays taken 24 months postoperatively showed solid bone union of the clavicle, coracoid process, and acromion

In this study, among the 27 patients with fractures of both the clavicle and coracoid process of the scapula on the same side, 6 had medial clavicle fractures, 12 had clavicle shaft fractures, and 9 had distal fractures. To the best of our knowledge, no previous studies have reported patients with fractures of the medial clavicle and ipsilateral coracoid process (Fig. 3), and fractures of the medial clavicle and shaft may be more common that previously thought.

In this study, 24 patients (88.8%) sustained injuries from high-energy trauma, and 19 patients (70.4%) had additional injuries. The specific injury mechanism remains unclear. One theory suggests that when an injury occurs, the force is transferred directly from above downward to the anterolateral aspect of the shoulder girdle, resulting in a distal clavicle fracture. Subsequently, the downward force causes the clavicle shaft to be pulled down, impacting the first rib and resulting in a clavicle shaft fracture. This impact also causes fractures of the coracoid and scapular neck [10]. Conversely, another theory posits that the force is directly applied to the lateral aspect of the shoulder girdle, resulting in a clavicle fracture. This force causes the clavicle fracture to shift upward, pulling the coracoclavicular ligament and causing an avulsion fracture of the coracoid process [12].

In general, Eyres type I to III coracoid fractures are frequently treated with nonoperative management, whereas type IV and V fractures are usually managed with internal fixation [22]. Additionally, the coracoid is part of the superior shoulder suspensory complex (SSSC). According to the theory proposed by Goss [28] in 1993, isolated lesions of the SSSC are relatively common and do not significantly alter the anatomical and functional stability of the shoulder ring. However, lesions affecting two or more points are much rarer and potentially unstable, possibly requiring operative stabilization [29]. Therefore, in this study, 22 patients were treated operatively to restore a stable connection between the clavicle and scapula, allowing for early physiotherapy. The clavicle fracture was first reduced and fixed with a 3.5 mm locking plate. Once the clavicle was stabilized, an indirect, satisfactory reduction of the coracoid process fracture was able to be achieved, and additional surgical fixation was no longer necessary because the fracture would likely consolidate in the correct position. However, if a reduction of the coracoid fracture was not achieved after reducing the clavicle, or if the fracture extended to the glenoid, further reduction and additional fixation were necessary.

Although several surgical techniques and implants for coracoid process fixation have been described, including screws with washers, cannulated screws, tubular plates, and reconstruction plates, metallic screw fixation (with or without washers) from the coracoid process into the scapular neck is generally considered the standard operative approach [19, 30]. Hill et al. [27] advocated for the use of double screw fixation if the fracture line extends to the base of the coracoid process. They also recommended using screws between 30 and 45 mm in length, with 15° of medial angulation and 30–40° of posterior angulation. The coracoid process, however, is located very close to the brachial plexus nerve, axillary artery, and vein. Additionally, it is an irregular bony process with numerous muscle and ligament attachments, making screw placement particularly challenging. With the introduction of robot-assisted technology at the authors’ institution in recent years, we used this technology to plan screw channel direction intraoperatively for three patients in this study.

This study has limitations, including its single-center, retrospective design; short follow-up period; and lack of a control group and biomechanical experiments. However, because of the rarity of these injuries, the purpose of this study was not to compare different treatment options but to report the incidence, characteristics, and outcomes of this type of injury. In future studies, more cases will be included and different fixation strategies will be compared.

## Conclusions

Ipsilateral concomitant fractures of the clavicle and coracoid process of the scapula are very rare. Clavicle fractures can occur at any location, and fractures of the shaft and medial end are more common than previously thought. A computed tomography scan is recommended when there is a high clinical suspicion of a coracoid process fracture. Treatment should be tailored to each injury pattern, with displaced fractures generally responding well to operative management. Additionally, the coracoid process may not require fixation if satisfactory indirect reduction is achieved following clavicle fixation.

## Data Availability

The datasets used and/or analyzed during the current study are available from the corresponding author on reasonable request.

## References

[1] Matsubara Y, Nakamura Y, Sasashige Y, Yokoya S, Adachi N (2023) Long-term conservative treatment outcomes for midshaft clavicle fractures: a 10-to-30-year follow-up. J Orthop Surg Res 18(1):952. 10.1186/s13018-023-04450-9 38082411 10.1186/s13018-023-04450-9 PMC10712139

[2] do Amaral FM, Malavolta EA, Silva FBAE, Garcia JC, da Silva Moura J, Assunção JH, Pecora JR (2023) Comparative study of patients with midshaft clavicle fracture fixed with a locked plate via an open versus percutaneous approach. Injury 54:110746. 10.1016/j.injury.2023.04.033 38143119 10.1016/j.injury.2023.04.033

[3] Frima H, van Heijl M, Michelitsch C, van der Meijden O, Beeres FJP, Houwert RM, Sommer C (2020) Clavicle fractures in adults; current concepts. Eur J Trauma Emerg Surg 46(3):519–529. 10.1007/s00068-019-01122-4 30944950 10.1007/s00068-019-01122-4

[4] Feng D, Jiang W, Kang X, Jiang Y, Zhu Y, Zhang J (2023) Simultaneous bilateral traumatic clavicle fractures: incidence, characteristics, and surgical outcomes. BMC Musculoskelet Disord 24(1):112. 10.1186/s12891-023-06228-w 36765310 10.1186/s12891-023-06228-w PMC9912484

[5] van Doesburg PG, El Saddy S, Alta TD, van Noort A, van Bergen CJA (2021) Treatment of coracoid process fractures: a systematic review. Arch Orthop Trauma Surg 141(7):1091–1100. 10.1007/s00402-020-03496-2 32507949 10.1007/s00402-020-03496-2

[6] Wu K, Wu XM, Zha XL, Wang QG (2020) Anatomic restoration of triple disruption of the superior shoulder suspensory complex: a case report and review of the literature. Orthop Surg 12(5):1526–1530. 10.1111/os.12764 32975039 10.1111/os.12764 PMC7670163

[7] Ogawa K, Ikegami H, Takeda T, Watanabe A (2009) Defining impairment and treatment of subacute and chronic fractures of the coracoid process. J Trauma 67(5):1040–1045. 10.1097/TA.0b013e318184205c 19680159 10.1097/TA.0b013e318184205c

[8] Ogawa K, Yoshida A, Takahashi M, Ui M (1997) Fractures of the coracoid process. J Bone Joint Surg Br 79(1):17–19. 10.1302/0301-620x.79b1.6912 9020438 10.1302/0301-620x.79b1.6912

[9] Doğar F, Dere Kİ, Gürbüz K, Topak D, Özdemir MA, Kuşcu B, Bilal Ö (2021) Rare coracoid fractures presenting with superior shoulder suspensory complex injury: a case series. Jt Dis Relat Surg 32(3):804–809. 10.52312/jdrs.2021.294 34842118 10.52312/jdrs.2021.294 PMC8650649

[10] Nanno M, Sawaizumi T, Ito H (2012) Double clavicular fractures associated with scapular neck and coracoid process fractures. J Orthop Surg (Hong Kong) 20(2):246–249. 10.1177/230949901202000223 22933689 10.1177/230949901202000223

[11] Ruchelsman DE, Christoforou D, Rokito AS (2010) Ipsilateral nonunions of the coracoid process and distal clavicle–a rare shoulder girdle fracture pattern. Bull NYU Hosp Jt Dis 68(1):33–37 20345361

[12] Sun CZ, Tao ZD, Mao WH, Wu XZ, Wu RW (2009) Treatment of clavicle fracture combined with coracoid process: a report of 3 cases. Zhongguo Gu Shang 22(5):346–347 19522390

[13] Allman FL Jr (1967) Fractures and ligamentous injuries of the clavicle and its articulation. J Bone Joint Surg Am 49(4):774–784 6026010

[14] Oe K, Gaul L, Hierholzer C, Woltmann A, Miwa M, Kurosaka M, Buehren V (2012) Operative management of periarticular medial clavicle fractures-report of 10 cases. J Trauma Acute Care Surg 72(2):E1-7. 10.1097/TA.0b013e31820d1354 21768908 10.1097/TA.0b013e31820d1354

[15] Cs NEER 2nd (1960) Nonunion of the clavicle. J Am Med Assoc 172:1006–1011. 10.1001/jama.1960.03020100014003 14426324 10.1001/jama.1960.03020100014003

[16] Eyres KS, Brooks A, Stanley D (1995) Fractures of the coracoid process. J Bone Joint Surg Br 77(3):425–428 7744929

[17] Ingoe HMA, Mohammed K, Malone AA, Beadle G, Sharpe T, Cockfield A, Lloyd R, Singh H, Colgan F (2023) Traumatic posterior sternoclavicular joint dislocation - current aspects of management. Injury 54(11):110983. 10.1016/j.injury.2023.110983 37634999 10.1016/j.injury.2023.110983

[18] Hu F, Han S, Liu F, Wang Z, Jia H, Wang F, Hu L, Chen J, Wang B, Yang Y (2022) A modified single-endobutton technique combined with nice knot for treatment of Rockwood type III or V acromioclavicular joint dislocation. BMC Musculoskelet Disord 23(1):15. 10.1186/s12891-021-04915-0 34980065 10.1186/s12891-021-04915-0 PMC8725473

[19] Tan Y, Li H, Wu J, Xue D, Pan Z (2023) Coracoid process fractures of the scapula treated by baseplate three-column glenoid fixation: a retrospective study of 24 patients. Med Sci Monit 29:e937933. 10.12659/MSM.937933 37032522 10.12659/MSM.937933 PMC10103615

[20] Feng D, Yang Y, Kang X, Heng L, Zhang J, Zhu Y (2023) Extra-articular locking plate and trans-articular clavicle hook plate for displaced medial clavicle fractures. Injury S0020–1383(23):00279–00286. 10.1016/j.injury.2023.03.023 10.1016/j.injury.2023.03.023 36964034

[21] Feng WL, Cai X, Li SH, Li ZJ, Zhang K, Wang H, Zhang J, Zhu YJ, Feng DX (2020) Balser plate stabilization for traumatic sternoclavicular instabilities or medial clavicle fractures: a case series and literature review. Orthop Surg 12(6):1627–1634. 10.1111/os.12726 32893491 10.1111/os.12726 PMC7767773

[22] Zhang L, Xiong L, He S, Liu J, Zhou X, Tang X, Fu S, Wang G (2022) Classification and morphological parameters of the coracoid process in Chinese population. J Orthop Surg 30(1):23094990211069696. 10.1177/23094990211069694 10.1177/23094990211069694 35041540

[23] Onada Y, Umemoto T, Fukuda K, Kajino T (2016) Coracoid process avulsion fracture at the coracoclavicular ligament attachment site in an osteoporotic patient with acromioclavicular joint dislocation. Case Rep Orthop 2016:9580485. 10.1155/2016/9580485 27493819 10.1155/2016/9580485 PMC4967432

[24] Goss TP (1996) The scapula: coracoid, acromial, and avulsion fractures. Am J Orthop 25(2):106–115 8640380

[25] Sharma N, Mandloi A, Agrawal A, Singh S (2016) Acromioclavicular joint dislocation with ipsilateral mid third clavicle, mid shaft humerus and coracoid process fracture - a case report. J Orthop Case Rep 6(2):24–27. 10.13107/jocr.2250-0685.414 27703932 10.13107/jocr.2250-0685.414 PMC5040563

[26] Ogawa K, Matsumura N, Yoshida A, Inokuchi W (2021) Fractures of the coracoid process: a systematic review. JSES Rev Rep Tech 1(3):171–178. 10.1016/j.xrrt.2021.04.008 37588963 10.1016/j.xrrt.2021.04.008 PMC10426686

[27] Hill BW, Jacobson AR, Anavian J, Cole PA (2014) Surgical management of coracoid fractures: technical tricks and clinical experience. J Orthop Trauma 28:e114–e122 24751608 10.1097/01.bot.0000435632.71393.bb

[28] Goss TP (1993) Double disruptions of the superior shoulder suspensory complex. J Orthop Trauma 7:99e106 8459301 10.1097/00005131-199304000-00001

[29] Ben-Ari E, Pines Y, Gordon D, Zuckerman JD, Petchprapa C, Virk MS (2022) Radiographic and clinical characterization of coracoid fractures: a retrospective cohort analysis. Eur J Orthop Surg Traumatol 32(8):1601–1607. 10.1007/s00590-021-03144-4 34628533 10.1007/s00590-021-03144-4

[30] Sun Z, Li H, Wang B, Yan J, Han L, Han S, Yang X, Zhao B (2021) A guideline for screw fixation of coracoid process base fracture by 3D simulation. J Orthop Surg Res 16(1):58. 10.1186/s13018-021-02203-0 33446228 10.1186/s13018-021-02203-0 PMC7809839

